# International Delphi-based consensus on the appropriate use and effect of Benzydamine hydrochloride in the treatment of sore throat

**DOI:** 10.1186/s12875-022-01901-w

**Published:** 2022-11-23

**Authors:** Desiderio Passali, Veronica Barat, Olga Cadevall, Hugo Miguel Freire, Ignazio Grattagliano, Ioan Gutu, Ralph Mösges, Andrey Pavlysh

**Affiliations:** 1 International Federation ORL Societies (IFOS) Executive Board members, Rome, Italy; 2 AOU Città della Salute e della Scienza di Torino, Regina Margherita Children’s Hospital, Turin, Italy; 3 Clínica Rotger, Quirónsalud, Palma de Mallorca, Spain; 4 Sociedade Portuguesa de Farmacêuticos dos Cuidados de Saúde, Coimbra, Portugal; 5 Italian College of General Practitioners and Primary Care, Florence, Italy; 6 General Practitioner, Vaslui, Romania; 7 Institute of Medical Statistics, and Computational Biology (IMSB), Medical Faculty, University at Cologne, Cologne, Germany; 8 North-Western State Medical University named after I.I. Mechnikov, Saint Petersburg, Russia

**Keywords:** Sore throat, Benzydamine, Oropharynx, Delphi-based consensus, General practitioner, Pharmacists, Symptomatic treatment of acute sore throat

## Abstract

**Background:**

Benzydamine hydrochloride is a locally-acting Non-Steroidal Anti-Inflammatory Drug (NSAID) with combined local anesthetic and analgesic properties, indicated for the symptomatic relief of pain in acute sore throat. The aim of this study was to obtain an European Consensus among pharmacists, general practitioners and pediatricians on the appropriate use of benzydamine hydrochloride in the treatment of sore throat.

**Methods:**

The authors developed a Delphi questionnaire organized into 15 statements focused on 4 topics: the mechanism of action of benzydamine, the benzydamine treatment in an adult patient and in a pediatric patient, and the advantages of benzydamine over other topical treatments. The survey was administered to a panel of to 320 participants including general practitioners, pediatricians, and pharmacists from 6 European countries (Italy, Germany, Portugal, Romania, Russia, and Spain), who rated their level of agreement or disagreement with each statement on a 6-point Likert scale. Consensus was predefined as more than 66% of the panel agreeing/disagreeing with each statement.

**Results:**

Panelists’ agreement on statements was very high. Consensus was reached for all 15 statements in the Delphi survey, with more than 98% positive agreement on topic 4, suggesting a shared view among European healthcare professionals (HCPs) about the advantages of benzydamine over other topical treatments. A strong consensus (> 99%) was reached for all the statements of topic 1 regarding the mechanism of action of benzydamine, except for its anesthetic properties (79%). Strong agreement was reached for all statements in topics 2 and 3 regarding the treatment of acute sore throat symptoms in the adult and pediatric patient, except for one on the efficacy of benzydamine in preventing post-operative sore throat, for which it was 67%.

**Conclusion:**

Because all relevant publications on benzydamine are dated and there are no recommendations on its use for the symptomatic treatment of sore throat in European guidelines, this Delphi-based international consensus may be important in reinforcing the appropriate use and effect of benzydamine in the treatment of sore throat among health care professionals.

## Introduction

Acute sore throat is a symptom often caused by an inflammatory process in the pharynx, tonsils, or nasopharynx, and is a common reason for consulting primary care physicians, pediatricians, and otolaryngologists (ENT) [1–3].

On average, adults have two to four and children six to eight upper respiratory tract infections per year, usually during the colder months [4, 5]. Most cases of acute sore throat are self-limiting, have a viral etiology, and occur as part of a common cold. In addition to viruses, bacterial pathogens can also cause pharyngeal infection, the most common being *Streptococcus pyogenes* (group A beta-hemolytic streptococcus) detected in 20% of cases, depending on seasonal and regional conditions and age [6]. Groups C or G beta-haemolytic streptococci, *Mycoplasma pneumoniae*, and *Chlamydia pneumoniae* have also been identified as pathogens [7]. The non-infective causes of sore throat are usually due to environmental variations, such as temperature changes, low humidity, second hand smoking, air pollution and a reaction to allergens [8].

Most patients with acute sore throat experience symptoms that include pain or difficult swallowing, redness and swelling of the throat, burning and irritation of the throat and cough [9]. Because only a small proportion of sore throat cases are caused by a bacterial infection, antibiotics are ineffective in most cases and are not recommended as the first line of treatment for acute sore throat in the EU [10–12].

Benzydamine hydrochloride is a locally-acting Non-Steroidal Anti-Inflammatory Drug (NSAID) indicated for the symptomatic relief of pain in acute sore throat, available in all European countries as an over-the-counter drug (except in Turkey) marketed under the brand name “Tantum Verde” [13]. In addition to its anti-inflammatory activity, benzydamine has antiseptic properties and combined local anesthetic and analgesic effects that in the topical route can be fully exploited and transformed into competitive advantages over other NSAIDs [14–16].

The effectiveness of benzydamine in relieving throat pain and dysphagia has been shown in different studies. Compared with placebo, benzydamine showed a significantly better reduction of pain with a more rapid decrease of pain severity [17–20]. In fact, the elective therapeutic use is the topical control of acute inflammation and pain. Benzydamine has its target indication in the symptomatic treatment of pain and irritative /inflammatory conditions of the oropharynx (gingivitis, stomatitis, pharyngitis), even when due to dental causes [13, 21]. Benzydamine has been studied and found effective for the treatment of oral mucositis and oral ulcers, as well as for the prevention of post-operative sore throat after endotracheal intubation [22–25].

The three main activities of benzydamine, anti-inflammatory, local analgesic, and anesthetic, all contribute to a highly specific and targeted efficacy by reducing symptoms related to a local inflammatory state. The ability of benzydamine to concentrate in inflamed tissues, with low systemic exposure, is a clear advantage that limits potential systemic side effects [13].

The aim of this study was to obtain an International Consensus among pharmacists, general practitioners and pediatricians on the appropriate use of benzydamine hydrochloride in the treatment of sore throat. This Delphi-based consensus of a European expert panel may be important in strengthening the use of benzydamine in the treatment of sore throat among healthcare professionals (HCPs) and filling the gap in guidelines at the European level. This gap is due to the fact that there are no recent studies on benzydamine and therefore all relevant publications are dated and do not fall within the analysis period considered in the guidelines.

Several projects and clinical trials are currently underway to collect new data on the use of benzydamine in the treatment of sore throat and to thoroughly investigate its mechanisms of action.

## Study design and methods

### Delphi method

The Delphi method is a structured technique designed to obtain a consensus opinion from a panel of experts in areas where scientific evidence is weak or necessary [26–28].

The Delphi method involves the repeated administration of questionnaires and allows obtaining both individual opinions and stimulating a debate around the research topic. The primary goal of Delphi is to narrow the range of responses and to reach a convergence of opinion or to facilitate the achievement of as much consensus as possible.

In this manuscript, the consensus process consisted of a one-step web-based Delphi method, which took place between November 2021 and January 2022.

### Delphi questionnaire preparation and administration

The survey was developed by a board of eight experts from different specialties (two general practitioners, one pharmacologist, one pharmacist, two pediatricians, and two otolaryngologists) and countries (Italy, Germany, Portugal, Romania, Russia and Spain), identified here as Key Opinion Leaders (KOLs) in the field of sore throat.

After reviewing the published literature on benzydamine, the KOLs met in a first virtual meeting to discuss the main areas of interest and identified four major topics: the mechanism of action of benzydamine, the benzydamine treatment in an adult patient and in a pediatric patient, and the advantages of benzydamine over other topical treatments. Each topic was sub-divided in a variable number of statements corresponding to items where greater need of clarification and debate existed.

Subsequently, the questionnaire was distributed via an online platform to 320 participants including general practitioners, pediatricians, and pharmacists from the following countries: Italy, Germany, Portugal, Romania, Russia, and Spain. Panelists for the Delphi questionnaire were chosen based on certain characteristics: with at least 5 years of experience in the field of sore throat, working in a hospital or clinic, or as freelancers, also considering a possible gender balance (50% men and 50% women). The panelists were identified with the support of an Italian research marketing company operating in the pharmaceutical industry and quality-certified (ISO 20252), which selected clinicians from its database based on the profile defined by the KOLs and invited them to answer the Delphi questionnaire. Voting was anonymous, and no compensation was given to any of the identified participants.

For each statement of the questionnaire, the panelists were invited to express the level of their agreement or disagreement according to the following 6-point Likert scale: 1 = strongly disagree, 2 = disagree, 3 = slightly disagree, 4 = slightly agree, 5 = agree and 6 = strongly agree. Results were expressed as a percentage of respondents who scored each item as 1, 2 and 3 (disagreement) or as 4, 5 or 6 (agreement). An item achieved consensus, when the sum of items 1, 2 and 3 (negative consensus) or 3, 4, and 5 (positive consensus) reached 66%. No consensus was reached, when the sum of the responses for a negative consensus or a positive consensus was below 66% [27, 28].

Descriptive analysis was performed to summarize the results.

### Compliance with ethics guidelines

The study was based on a survey that does not involve the participation of human subjects nor patient data management, so this study did not require ethical approval nor prior approval of the study protocol. All experts involved in the Delphi survey were informed of the study’s objectives and the possibility of publishing the results in a peer-reviewed article. The participation was voluntary. The panelists expressed their consent to participate in the survey after logging into the secure online survey platform via credentials, by actively clicking on the appropriate box.

## Results

### Information on the participants

Three hundred and twenty panelists among general practitioners, pediatricians, and pharmacists were identified in the following countries: Italy, Germany, Portugal, Romania, Russia and Spain.

Three hundred and two respondents from these 6 European countries out of 320 invited panelists (response rate 94.4%) completed the Delphi questionnaire with a homogeneous international distribution (51 from Italy, 50 from Germany, 50 from Portugal, 49 from Romania, 51 from Russia and 51 from Spain). The panelists were 101 general practitioners, 101 pediatricians, and 100 pharmacists.

### Degree of consensus in the Delphi process

Panelists’ agreement on statements was very high. Consensus was reached for all 15 statements in the Delphi survey (100%), with more than 98% positive agreement on topic 4, suggesting a shared view among European HCPs about the advantages of benzydamine over other topical treatments.

A strong consensus (> 99%) was reached for all the statements of topic 1 regarding the mechanism of action of benzydamine, except for its anesthetic properties (79%).

Strong agreement was reached for all statements in topics 2 and 3 regarding the treatment of acute sore throat symptoms in the adult and pediatric patient, except for one on the efficacy of benzydamine in preventing post-operative sore throat, for which it was 67%.

Table 1 summarizes the statements and items and presents the percentage agreement/disagreement for each based on the responses of the 302 panelists.

**Table 1:** Results of the Delphi survey

Statement	Consensus score	
	Disagreement (score 1–3) (%)	Agreement (score 4–6) (%)
**Topic 1 - Mechanism of action**		
**1.** The different modes of action of Benzydamine in the treatment of sore throat are:		
**(1.1)** Anti-inflammatory	0%	100%
**(1.2)** Anesthetic	21%	79%
**(1.3)** Analgesic	1%	99%
**(1.4)** Antiseptic	1%	99%
**(2)** The anti-inflammatory activity of Benzydamine has been related to its ability to inhibit the release of pro-inflammatory cytokines (TNFa, IL-1β, and MCP-1), without affecting other anti-inflammatory factors, thus not presenting the side effects characteristic of aspirin-like drugs.	1%	99%
**(3)** Differently from other NSAIDs, Benzydamine exhibits local anesthetic properties by modulating neuronal sodium-dependent excitability so when applied topically in clinically relevant concentrations it exerts its effect on pain.	1%	99%
**Topic 2 – Adult patient**		
**(4)** According to my experience, the decision to prescribe/recommend Benzydamine is based on the presence of the following symptoms:		
**(4.1)** Ache	5%	95%
**(4.2)** Itchy throat	25%	75%
**(4.3)** Dry throat	29%	71%
**(4.4)** Difficulty in swallowing	6%	94%
**(4.5)** Redness of the throat	9%	91%
**(4.6)** Cough	9%	91%
**(5)** In the absence of bacterial infections, antibiotics should not be used to provide symptomatic relief in sore throat. Either non-steroidal anti-inflammatory drugs or paracetamol are recommended for relief of acute sore throat symptoms.	1%	99%
**(6)** I recommend Benzydamine for the short-term symptomatic treatment of acute sore throat.	2%	98%
**(7)** According to my experience, Benzydamine is preferred over other local analgesics to reduce symptoms of acute sore throat.	12%	88%
**(8)** Benzydamine is effective on preventing post-operative sore throat.	33%	67%
**Topic 3 – Pediatric patient**		
**(9)** According to my experience, the decision to prescribe/recommend Benzydamine is based on the presence of the following symptoms:		
**(9.1)** Ache	4%	96%
**(9.2)** Itchy throat	19%	81%
**(9.3)** Dry throat	23%	77%
**(9.4)** Difficulty in swallowing	7%	93%
**(9.5)** Redness of the throat	6%	94%
**(9.6)** Cough	9%	91%
**(10)** Ibuprofen or paracetamol is recommended for the relief of general pain or fever associated with acute pharyngitis.	1%	99%
**(11)** I recommend Benzydamine (mouthwash or losenges or spray) for the short-term symptomatic treatment of acute sore throat.	4%	96%
**(12)** According to my experience, Benzydamine (mouthwash or losenges or spray) is preferred over other local analgesics to reduce symptoms of acute sore throat.	15%	85%
**Topic 4 – Advantages of Benzydamine over other topical treatments**		
**(13)** Benzydamine may quickly reduce the pain of acute sore throat.	0%	100%
**(14)** The mechanisms of action of Benzydamine, especially its local anesthetic activity, according to my experience, constitute a plus compared to other topically administered drugs.	0%	100%
**(15)** The combined anti-inflammatory activity of Benzydamine with its local anesthetic and analgesic properties, and its antiseptic activity, according to my experience, constitute an advantage over other topically administered drugs.	2%	98%

## Discussion

### Topic 1 - mechanism of action (statements 1, 2 and 3)

The consensus on benzydamine’s mechanisms of action was nearly unanimous, except for its anesthetic action (79%): panelists recognized benzydamine’s anti-inflammatory activity (100%) and its analgesic and antiseptic properties (99%). The most recognized feature of benzydamine is its anti-inflammatory characteristic [29].

The panelists strongly agreed (99%) that, unlike NSAIDs that act by inhibiting prostaglandin synthesis, the anti-inflammatory activity of benzydamine is related to its ability to inhibit the release of pro-inflammatory cytokines (TNFα, IL-1β, and MCP-1), without affecting other anti-inflammatory cytokines (IL-10, IL-1ra). This statement is supported by a high level of scientific evidence. The studies of Sironi et al. confirmed that the anti-inflammatory activity of benzydamine is carried out by the selective inhibition of pro- versus anti-inflammatory cytokines [30, 31].

The less positive consensus on benzydamine’s anesthetic mode of action may be explained by the fact that the symptomatology for which benzydamine use is indicated is related to an inflammatory condition; therefore, an anti-inflammatory rather than anesthetic response is expected. Thus, it is likely that the anesthetic activity of benzydamine is less well known among the pharmacists and primary care physicians who participated in this survey because the drug is presented as an anti-inflammatory compound.

However, the use of benzydamine as a local anesthetic is widespread, especially after procedures involving the oral cavity (such as tonsillitis) or after dental extraction, because of its transient topical anesthetic effect that provides symptomatic relief.

Although the anesthetic property of benzydamine achieved a slightly strong agreement (79%), panelists acknowledge that benzydamine also exhibits anesthetic activity by modulating neuronal excitability (99%). This statement reflects the high level of scientific evidence that has confirmed that benzydamine is a blocker of Na_v_ channels expressed in sensory neurons, and that it can be a modulator of nociceptor excitability. In fact, benzydamine attenuates nociceptor excitability and local transmission of the pain stimulus by preventing the generation and propagation of action potentials through the block of voltage-gated Na + channels [32]. In addition, benzydamine has been shown to share with local anesthetics an aromatic (hydrophobic) ring structure linked to a basic tertiary amine group (hydrophilic) by a short alkyl chain. Therefore, benzydamine, like local anesthetics, reversibly blocks nerve conduction when applied topically in proper concentrations [33].

The local anesthetic activity of benzydamine has been shown to be extremely useful in the treatment of painful conditions of the mouth and throat, primarily due to rapid pain relief [14].

### Topic 2 – adult patient (statements 4,5,6,7 and 8)

A high positive consensus to prescribe/recommend benzydamine was achieved in presence of the symptoms ache (95%), difficulty in swallowing (94%), redness of the throat (91%) and cough (91%), while lower agreement was observed in relation to itchy throat and dry throat (75 and 71%, respectively). However, physicians and pharmacists are likely to consider for dry and itchy throat some type of syrup or other type of compound, for example, cetylpyridinium chloride and benzocaine instead of benzydamine. This may explain a lower level of agreement for these two symptoms for which benzydamine may not be considered the first choice treatment.

There was almost unanimous consensus in recommending analgesics such as paracetamol and NSAIDs to relieve the symptoms of acute sore throat (99%). This consensus is in line with European guidelines that recommend both ibuprofen or paracetamol to relieve symptoms of acute sore throat [10, 34, 35].

In addition, because there is no convincing evidence of benefit from antibiotic therapy as primary treatment for sore throat and the superiority of antibiotics over simple analgesics is marginal in reducing the duration or severity of symptoms [36, 37], these guidelines do not encourage the use of antibiotics to provide symptomatic relief in sore throat. In view of increases in healthcare-acquired infections and antibiotic resistance in the community, antibiotics for minor self-limiting illness should not be prescribed. The European Society for Clinical Microbiology and Infectious Diseases guidelines do not recommend the use of antibiotics in patients with a less severe presentation of sore throat, (e.g., Centor criteria 0–2) to relieve symptoms [10]. According to the German guidelines, sore throat (even of bacterial etiology) is not a general indication for antibiotic administration. Therefore, the updated guideline explicitly supports foregoing antibiotic treatment in the German healthcare context, even in cases of strong clinical suspicion of bacterial tonsillopharyngitis [34]. In this context, benzydamine as an effective analgesic for acute sore throat symptoms could reduce the prescription of unnecessary antibiotics.

Ninety-eight percent of participants strongly agreed to recommend benzydamine for short-term symptomatic treatment of acute sore throat, and 88% would prefer benzydamine to other local analgesics to reduce symptoms of acute sore throat. These findings represent clinical practice and real-life data from the countries that participated in the study, as no recommendations on the use of benzydamine for the symptomatic treatment of sore throat appear in European guidelines on the management of acute sore throat [10, 34, 38].

A Canadian study examined the clinical effectiveness of benzydamine oral rinse (0.15%) for pain relief in acute sore throat and conducted a literature search on documents, published between 2008 and 2018, informing the use of benzydamine for the symptomatic treatment of acute sore throat [39]. In this review, neither guidelines nor relevant studies on the clinical efficacy of benzydamine for pain relief in acute sore throat were identified, with the exception of a single evidence-based guideline developed by the Scottish Intercollegiate Guidelines Network (SIGN), and published in 2010, which provides recommendations for pain management in acute sore throat, including benzydamine [35]. However, with respect to adjunctive therapy including benzydamine topical agents, the guideline did not provide a recommendation due to the insufficiency of evidence.

A weak positive consensus, almost close to no consensus (67%), was reached with respect to the statement about the efficacy of benzydamine in preventing post-operative sore throat. In this case, the responses may have been influenced by the different indications for benzydamine in the European countries involved. Indeed, benzydamine for oromucosal use is indicated in the relief of pain and irritation of the mouth and throat, but in some eastern countries there is also an indication for post-operative sore throat. Consequently, this clinical indication is better known by specialist involved in surgical procedures respect to the GPs and pharmacists, who expressed low levels of agreement.

However, this statement is supported by scientific evidence and clinical trials that confirmed the effect of benzydamine hydrochloride on the prevention of postoperative sore throat [40–42].

### Topic 3 – pediatric patient (statements 9, 10, 11 e 12)

Also in the pediatric field, benzydamine is indicated for the topical treatment of sore throat symptoms. A common and positive consensus was reached for the treatment of sore throat symptoms with benzydamine, with slightly less agreement for itchy throat and dry throat in the pediatric patient as well. A high positive consensus was achieved for the symptoms ache (96%), difficulty in swallowing (93%), redness of the throat (94%) and cough (91%), while lower agreement was observed in relation to itchy throat and dry throat (81 and 77%, respectively). It is possible that for these two symptoms other types of local products such as honey or candies are perhaps preferred in pediatric setting.

A large consensus was reached on the statement that ibuprofen or paracetamol is recommended for the relief of general pain or fever associated with acute pharyngitis (99%). This statement is supported by a high level of evidence. A systematic review showed that ibuprofen and paracetamol are more effective than placebo for reducing acute sore throat symptoms in children [43]. Other systematic reviews have confirmed the efficacy and safety of single doses of ibuprofen and paracetamol for the short-term treatment of pain or fever in pediatric populations [44, 45].

Moreover, the recommendation on the use of ibuprofen or paracetamol to relieve pain or fever associated with discomfort in children with acute pharyngitis is contained in the European guidelines on the management of sore throat [10, 34, 46, 47].

Ninety-six percent of panelists would recommend benzydamine (mouthwash or lozenges or spray) for short-term symptomatic treatment of acute sore throat, and 85% would prefer benzydamine to other local analgesics to reduce symptoms. These responses indicate that most respondents would recommend benzydamine for short-term symptomatic treatment of acute sore throat, but that some would prefer a natural product or other supplements for acute sore throat in the pediatric setting.

As with the adult patient, these are important findings because there are no evidence-based guidelines and relevant studies on the use of benzydamine in the symptomatic treatment of acute sore throat in the pediatric population. Only a placebo-controlled clinical trial performed in 146 children (aged 5 to 17 years) with sore throats showed that benzydamine 0.15% mouthwash was significantly better (*p* < 0.05) than placebo in all efficacy parameters, namely the Children’s Throat Pain Thermometer (VAS 0 to 200 mm), the Children’s Throat Relief Scale (Scale 0 to 4 units), and the Nurses’ Pain Change Scale (VAS 0 to 100 mm) [48].

### Topic 4 – advantages of Benzydamine over other topical treatments (statement 13, 14 and 15)

The totality of the panelists agreed with the statement that benzydamine may quickly reduce the pain of acute sore throat (100%).

There was full agreement that benzydamine’s mechanisms of action, especially its local anesthetic activity, constitute a plus compared to other topically administered drugs (100%).

A large consensus was reached on the statement that the combined anti-inflammatory activity of benzydamine with its local anesthetic and analgesic properties, and its antiseptic activity are an advantage over other topically administered drugs (98%).

Panelists’ agreement with these statements was very high, suggesting a shared view among European HCPs about the advantages of benzydamine over other topical treatments.

This consensus is important to strengthen the use of benzydamine in the treatment of sore throat among HCPs, especially because there are no recent studies on benzydamine and the clinical trials for registration of this product were performed in the 1980s and 1990s and therefore all relevant publications are dated.

Several projects and clinical trials are underway to collect new clinical data on the use of benzydamine in the treatment of sore throat and to thoroughly investigate its mechanisms of action [49, 50].

## Conclusions

Although classified as an NSAID, benzydamine demonstrates mechanisms of action that differ from those of traditional aspirin-like NSAIDs, promoting it as an effective locally-acting NSAID with local anesthetic and analgesic properties.

The panelists’ agreement with these statements was very high, suggesting an excellent knowledge of the mechanisms of action of benzydamine and its therapeutic use in the symptomatic treatment of acute sore throat in both adult and pediatric patients. The panelists recognize that the combined anti-inflammatory activity of benzydamine with its local anesthetic and analgesic effects, and its antiseptic property constitute an advantage compared to other topically administered drugs, as these activities all contribute to a highly specific and targeted efficacy, reducing symptoms related to a local inflammatory state. However, it seems from these results that it is necessary to emphasize the local anesthetic activity of benzydamine, which is extremely useful in the treatment of mouth and throat painful conditions, especially for its rapid pain relief.

This European expert panel of pharmacists, general practitioners, and pediatricians strongly agree in recommending benzydamine for the short-term local and symptomatic treatment of acute sore throat and in preferring benzydamine to other local analgesics to reduce the symptoms.

Because all relevant publications on benzydamine are dated and there are no recommendations on its use for the symptomatic treatment of sore throat in European guidelines, this Delphi-based international consensus may be important in reinforcing the appropriate use and effect of benzydamine in the treatment of sore throat among health care professionals.

## Data Availability

The data that support the findings of this study are available from the corresponding author, DP, upon reasonable request.
